# A Comprehensive Review of Lab-Scale Studies on Removing Hexavalent Chromium from Aqueous Solutions by Using Unmodified and Modified Waste Biomass as Adsorbents

**DOI:** 10.3390/toxics12090657

**Published:** 2024-09-08

**Authors:** Manikant Tripathi, Sukriti Pathak, Ranjan Singh, Pankaj Singh, Pradeep Kumar Singh, Awadhesh Kumar Shukla, Sadanand Maurya, Sukhminderjit Kaur, Babita Thakur

**Affiliations:** 1 Biotechnology Program, Dr. Rammanohar Lohia Avadh University, Ayodhya 224001, Uttar Pradesh, India; sukritipathak24@gmail.com (S.P.); singhpankaj0984@rediffmail.com (P.S.); 2 Department of Microbiology, Dr. Rammanohar Lohia Avadh University, Ayodhya 224001, Uttar Pradesh, India; ranjan.singh13@gmail.com; 3 Department of Biochemistry, Dr. Rammanohar Lohia Avadh University, Ayodhya 224001, Uttar Pradesh, India; pkbt99@gmail.com; 4 Department of Botany, K.S. Saket P.G. College, Ayodhya 224001, Uttar Pradesh, India; awadhkshukla@gmail.com (A.K.S.);; 5 Department of Biotechnology, Chandigarh University, Mohali 140413, Punjab, Indiababitathakur18may@gmail.com (B.T.)

**Keywords:** toxic chromium, adsorption, environmental pollution, waste biomass, sustainability

## Abstract

Anthropogenic activities and increasing human population has led to one of the major global problems of heavy metal contamination in ecosystems and to the generation of a huge amount of waste material biomass. Hexavalent chromium [Cr(VI)] is the major contaminant introduced by various industrial effluents and activities into the ecosystem. Cr(VI) is a known mutagen and carcinogen with numerous detrimental effects on the health of humans, plants, and animals, jeopardizing the balance of ecosystems. Therefore, the remediation of such a hazardous toxic metal pollutant from the environment is necessary. Various physical and chemical methods are available for the sequestration of toxic metals. However, adsorption is recognized as a more efficient technology for Cr(VI) remediation. Adsorption by utilizing waste material biomass as adsorbents is a sustainable approach in remediating hazardous pollutants, thus serving the dual purpose of remediating Cr(VI) and exploiting waste material biomass in an eco- friendly manner. Agricultural biomass, industrial residues, forest residues, and food waste are the primary waste material biomass that could be employed, with different strategies, for the efficient sequestration of toxic Cr(VI). This review focuses on the use of diverse waste biomass, such as industrial and agricultural by-products, for the effective remediation of Cr(VI) from aqueous solutions. The review also focuses on the operational conditions that improve Cr(VI) remediation, describes the efficacy of various biomass materials and modifications, and assesses the general sustainability of these approaches to reducing Cr(VI) pollution.

## 1. Introduction

With increasing global population and industrialization, the resultant population poses a significant risk to our ecosystem, which comprises living beings and the environment. Hazardous chemicals, including heavy metals, are released into the environment by several industries, including tanneries, paper mills, and others, that have detrimental effects on the environment [1,2,3,4,5]. Elements with a density greater than 5 g/cm^3^ are classified as heavy metals [4]; even at low concentrations, these elements can be dangerous [5]. According to Fan et al. [6], heavy metals are hazardous, non-biodegradable, and present in the environment, posing several risks to various living things. Hexavalent chromium Cr(VI) is regarded as toxic heavy metal since it poses a major risk to both the environment and human health [7,8]. There are many different oxidation states of the heavy metal chromium. The most stable forms of the metal are trivalent chromium Cr(III) and Cr(VI); Cr(VI) is widely distributed in a variety of environments [9,10,11]. According to Rakhunde et al. [12], Cr(VI) can be found in natural waters either as an oxyacid (H_2_Cr_2_O_7_/H_2_CrO_4_) or as oxyanions, which include HCrO_4_^−^, CrO_4_^2−^, HCr_2_O_7_^−^, Cr_2_O_7_^2−^. According to researchers [9,13], Cr(VI) has higher mutagenic and hazardous effects compared to Cr(III).

On the other hand, Cr(III) is essential for human metabolism, helping to maintain levels of cholesterol, triglycerides, and glucose [14,15,16]. However high concentrations of Cr can have negative consequences [9,17]. Given that Cr(VI) is more harmful than Cr(III), it is imperative to remove this from the ecosystem using a variety of sustainable techniques [17,18,19]. Human disorders may be caused by the presence of Cr(VI) in the food chain. When plants are grown close to chromium-polluted areas, Cr(VI) has adverse effects on the plants [20,21]. According to Singh et al. [22] and Brusseau and Artiola [23], the pollutant may have different types of toxic effects on living forms. Some legislative rules have been linked to the monitoring and release of chromium across many sectors due to the grave health risks associated with this element. A maximum limit of 0.05 mg/L for Cr(VI) discharge has been imposed by the United States Environmental Protection Agency (US EPA) for wastewater discharge from industrial sectors [9,23]. The limit for Cr in drinking water in India is 0.05 mg/L, while for treated wastewater from industrial discharge, it is 0.1 mg/L, according to the regulatory body [9,24].

The WHO (World Health Organization) states that 50 µg/L, is the standard for drinkable water [25]. According to Agarwal et al. [26], some physical and chemical techniques, including solvent extraction, ion exchange, electrochemical reduction/oxidation, reverse osmosis, precipitation, and photo-catalysis, have been used for chromium remediation. Nevertheless, these approaches are rather expensive, require a high level of skill and meticulous procedure control, and are not able to fully remove chromium [9,11]. However, due to its high efficacy, affordability, ease of use, and versatility in removing contaminants from contaminated systems, the adsorption strategy is gaining favor among researchers as a preferable method [27,28].

Many adsorbents, including activated carbon, biochar, and agricultural wastes, have been used for the remediation of heavy metals [29,30,31]. As a result, there is a need for adsorbents that can effectively remove chromium from wastewater while also being economically viable [2]. One of the best methods for treating water is the adsorption of Cr(VI) by sustainable and affordable adsorbents, which has several benefits, such as reusable properties and zero secondary contamination, which reduce potential drawbacks [32,33,34].

A variety of natural adsorbent materials have been used for different types of environmental pollutants [34,35,36,37,38,39,40]. In one study, activated banana peel was applied by Khalil et al. [41], while alkaline-hydrolyzed *Garcinia kola* shell was employed by Popoola et al. [42] to remove Cr(VI) from contaminated water. The emergence of waste material biomass as a potent adsorbent for the sequestration of Cr(VI) from contaminated wastewater was made possible by these and other investigations. Although several studies are available that claim the effectiveness of different biomass materials in Cr(VI) removal, still there is a lack of reports demonstrating the scale-up efficiency of their techniques in field trials. There is also a lack of studies in the literature on economic analyses for integrating these techniques into existing treatment strategies.

There are several methods, including physical, chemical and biological methods, that have been employed for the remediation of Cr(VI) from the polluted aquatic environment. However, the adsorption of Cr(VI) on a waste material biomass is a cost-effective treatment technology that is environmentally friendly. This review studied the toxicity levels of, and health hazards posed by, Cr(VI) accumulation, and analyzed the effectiveness of different waste biomass in Cr(VI) removal and their practical implications. This review also summarized mechanisms for Cr(VI) remediation employing unmodified and modified different waste material biomass in a sustainable manner.

## 2. Sources and Toxicity

Cr(VI) compounds are a broad class of compounds that have several properties, including robustness, stiffness, and resistance to corrosion [43]. As a result, they are used in a wide range of applications. Chromium trioxide, potassium chromate, ammonium chromate, sodium chromate, chromic acid, and calcium chromate are a few examples of compounds that include Cr(VI) [44]. The main commercial source of chromium is chromite (FeCrO_4_), which is extracted as a primary product [45]. Chromium is mostly used in chemistry, metallurgy, and refractories.

It is used as pigments for paints, inks, plastics, and anti-corrosion coatings (as zinc and lead chromate), textile dyes, leather tanning, wood preservatives, and stainless steel. According to Saha et al. [46], anthropogenic and natural sources are the two main sources of Cr(VI). Ultramafic igneous and metamorphic rocks, such as serpentinites and periodontitis, as well as the soil that was formed from them, are typically linked to natural sources of Cr(VI) in rocks, soils, and sediments. Mafic rocks may occasionally be involved in high chromium concentrations as well [47,48]. Cr(VI) can exist naturally as CrO_4_^2−^ in ground or surface water [46], but it can also be released into the atmosphere by volcanic eruptions, meteoric dust, forest fires, wind-blown sand, and volcanic eruptions [49]. According to Saha et al. [46], there is typically a greater mass deposition of chromium in metropolitan regions compared to isolated and rural places. According to the Agency for Toxic Substances and Disease Registry (ATSDR) [50], chrome plating has been found to be the main cause of atmospheric pollution by Cr(VI), generating high amounts of Cr(VI) annually. Surface water has an excessive level of chromium due to the textile, electroplating, and leather tanning industries [50,51]. Several researchers have studied metal contamination in different areas [52,53,54].

According to DesMarais and Costa [55], chromium exposure has occurred in humans due to the ingestion of contaminated water or contact with contaminants. Researchers [56] also studied the toxic effect of Cr(VI) on plants. According to several studies [9,57,58,59], Cr(VI) is a known carcinogen, mutagen, and toxicological agent. Studies have investigated the possibility that those who are exposed to chromium may become acutely unwell from it. Many exposure variables, such as chromium time duration, form, source, or dosage, can cause a variety of acute or moderate illnesses [50,51,52].

Three categories—acute, intermediate, and chronic—are based on the length and timing of Cr(VI) exposure [60,61]. (Cr(VI) exposure can occur primarily by ingestion, inhalation, or skin contact [60]. The exposure of Cr(VI) can have a wide range of harmful consequences on plants, animals, and other living things.

## 3. Toxicity of Cr(VI)

### 3.1. Toxic Effects on Human Health

**Figure 1:**
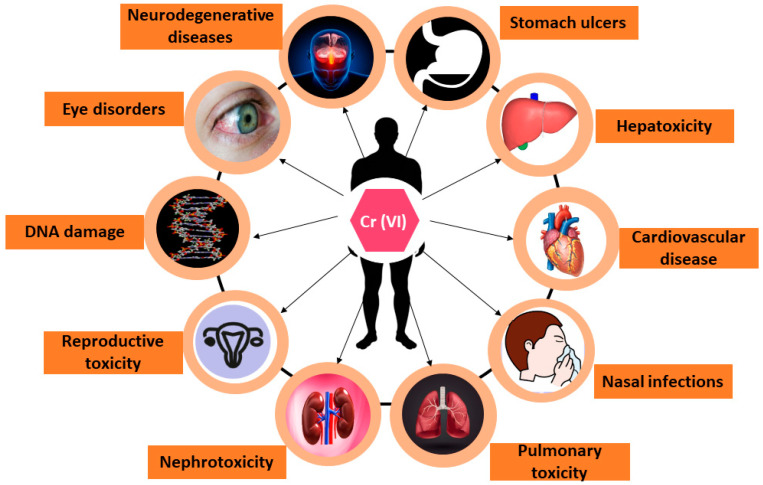
Toxicity of Cr(VI) in humans.

Cr(VI) is one of the major environmental pollutants. According to Nigam et al. [62], there are two possible ways that people might be exposed to Cr(VI): by inhalation (breathing) during an occupational exposure, or through food or water. In another study, Vendruscolo et al. [63] reported that Cr(VI) toxicity may have a variety of negative effects on health, including contact dermatitis, emphysema, internal bleeding, liver and kidney illness, hypersensitivity responses, and DNA damage, as shown in Figure 1. DesMarais and Costa [55] reported that the oxidative stress that Cr(VI) causes in cells might lead to the development of DNA adducts, cardiovascular disorders, and neurological illnesses. An individual may develop lung cancer or irritation/damage to their respiratory tract if they inhale large amounts of Cr(VI) [64]. Cr(VI) toxicity cause health hazards through adverse effects [60,64]. These findings suggest that exposure to Cr(VI) should be avoided for a healthy life, as it causes various types of illnesses in humans.

Compared to individuals of the same gender and age, the workers exposed to Cr(VI) in industries had an increased risk of oral, gastric, respiratory, throat, and prostate cancer, with an increased risk of cancer overall [65]. Similarly, Zhitkovich et al. [66] reported that Cr(VI) may produce unstable radicals such as hydroxide radicals, superoxide anions, thiol groups, and H_2_O_2_ and hence induce genotoxicity. Because of its considerable potential to induce a variety of malignancies, including sinus, bladder, nasal, stomach, paranasal, and nasal cancers, Cr(VI) has been classified by the International Agency for Research on Cancer (IARC) as a Group 1 occupational carcinogenic agent [67,68,69,70]. A range of respiratory conditions, including pneumonia, bronchitis, and reduced lung function, have also been noted in workers in chromium-related sectors [50]. Long-term exposure to Cr(VI) may also cause skin irritation and a burning feeling in the skin, which may lead to allergic contact dermatitis [44,50]. According to Li et al. [71], the high nephrotoxicity of Cr(VI) caused kidney damage, poor renal absorption, proteinuria, glycosuria, and renal dysfunction. Epidemiological investigations on female workers exposed to Cr(VI) have also shown reproductive system damage (developmental toxicity), with issues during pregnancy, delivery, and gestational development having been noted [72,73].

### 3.2. Toxic Effects on Plants

Cr(VI) is hazardous to plants and leads to the inhibition of various activities and may even cause complete damage to plants [74,75]. The toxicity of Cr(VI) reduces productivity, plant growth antioxidant enzyme activity, and photosynthetic pigments [76]. Due to high concentrations of Cr(VI) in soil, alfalfa seed germination was decreased, and bean development was also impacted [77]. Researchers have also noted a host of other symptoms in plants caused by Cr(VI) toxicity, including necrosis, chlorosis, lipid peroxidation, damage to root tissue, reduced enzyme activity, and impaired plant growth, including the reduced development of leaves, roots, and stems, the suppression of seed germination, and reduced photosynthesis [78,79,80].

### 3.3. Toxic Effects on Animals

Numerous research endeavors examined the detrimental impacts of Cr(VI) on fauna. Chen et al. [81], discovered that Cr(VI) may induce oxidative damage to roosters’ kidneys and damage to hens’ livers [82]. Similarly, Zhang et al. [83] noted that prolonged exposure to Cr(VI) in mice may result in colorectal cancer. In another study, Li et al. [84], investigated the changes in gut microbiota (gut microbial dysbiosis) brought on by prolonged exposure to Cr(VI) in chickens, while Yu et al. [85] reported on the increased suppression of immunity, digestion, and antioxidant capacity in *Channa asiatica* as a result of Cr(VI) exposure. Suljević et al. [86] used male Japanese quail (*Coturnix japonica*) as a toxicological model to evaluate Cr(VI) toxicity and found that it decreased the immune response and caused hypochromic and microcytic anemia.

### 3.4. Toxic Effects of Cr(VI) on Microbes

Microbes that are not tolerant to Cr(VI) may be at risk from higher concentrations of Cr(VI). Microbial alpha diversity is decreased in chromium-polluted areas [87,88]. Microbes that are resistant to Cr(VI) can, however, convert Cr(VI) to Cr(III) and withstand the toxicity [87,88,89]. Many researchers reported that different species of *Bacillus* were not only Cr(VI)-tolerant, but were also capable of remediating Cr(VI) [89,90]. In one study, Wyszkowska et al. [91] studied the toxic effects of Cr(VI) on the diversity of microorganisms found in soil. They reported that the presence of Cr(VI) in soil adversely impacted the population of organotrophic bacteria, indicating the toxic effects of the metal. In another study, Kang et al. [92] reported on the remediation of Cr(VI) by a potential bacterial isolate *Pseudomonas aeruginosa,* which was also tolerant to lead, cobalt, copper, mercury, and cadmium. In a recent research investigation, Ye et al. [90] also reported on Cr(VI) remediation a using *Bacillus mobilis* CR3 strain isolated from a Cr(VI)-polluted site. These studies revealed that the Cr(VI) posed negative effects on the growth of microorganisms; however, some microorganisms are tolerant to Cr(VI), and are also capable of removing such toxicants from contaminated environments.

## 4. Remediation Strategies for Cr(VI)

**Figure 2:**
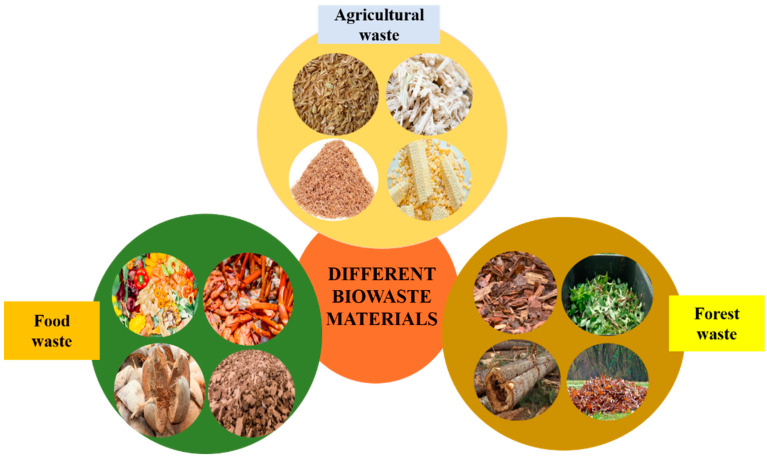
Sources and types of waste materials biomass.

The methods employed to remove Cr(VI) from contaminated sources can be classified as physical, chemical and biological methods. There are various disadvantages associated with physical and chemical methods of remediation such as limited capacity, hazardous secondary waste generation, low removal efficiency, and costly setups [9,11]. Biological techniques explore the natural abilities of microorganisms and plants to remove Cr(VI) by adsorption or transformation. Lately, a great deal of attention has been paid to the development of low-cost, high-efficiency adsorbents for adsorption and high chromium removal [93,94]. The adsorption and extraction of Cr(VI) from the environment could be accomplished using various, more environmentally friendly biosorption techniques using biomass waste materials, as shown in Figure 2.

### 4.1. Agricultural Residues

Agricultural waste materials biomass is a feasible resource found in ample quantities across the world [95,96]. Agricultural waste biomass has piqued interest in the generation of adsorbents and is preferred more than commercial adsorbents due to their several advantageous qualities, such as cost-efficient operation, and for their various bio-chemical properties [97,98,99]. Multiple agricultural residues can especially be obtained in an agri-abundant country like India [100]. Various agricultural residues can be used for Cr(VI) remediation such as rice husk, sugarcane bagasse, and corn cobs. The remediation of Cr(VI) utilizing agricultural biomass as adsorbents has been the subject of several investigations. Using rice husk ash and silica, Adelagun et al. [101] effectively removed Cr(VI) at pH values greater than 6. The highest adsorption capabilities of rice husk ash were reported to be 55.98 mg/g, and rice husk silica to be 67.45 mg/g, for the removal of Cr(VI). In 2020, Ogata et al. [102] conducted a study on the removal of Cr(VI) from wheat bran biomass. In another study, Yadav et al. [103] investigated the biosorption of Cr(VI) by rice husk biomass and found that the high amount of Cr(VI) could be adsorbed at a lower pH.

### 4.2. Food Waste

The Food and Agriculture Organization (FAO) estimates that 40% or more of the food produced is wasted [104]. In terms of the creation of fruit and vegetable waste, India ranks in fourth place, behind China, the United States, and the Philippines [100]. Fruit peels, for example, are a cheap and readily accessible food waste that may be used as an affordable adsorbent for the remediation of Cr(VI). Tie et al. [105] utilized food waste biomass for the formation biomass-derived biochar for the removal of Cr(VI) and found the highest adsorption rate of 20.4 mg/g at a pH of 2. Wang et al. [106] employed pomelo peel (PP) and ferric-chloride modified (FeCl_3_ modified) pomelo peel (FPP) for the adsorption of Cr(VI) and reported a 21.55 mg/g maximum Cr(VI) adsorption capacity by FPP and a 0.57 mg/g adsorption capacity by PP at a pH of 2 and a temperature 40 °C. In another study, Sahlabji et al. [107] employed pea peels as food waste for the preparation of environmentally friendly activated carbon to remediate Cr(VI), obtaining a maximum adsorption capacity of 480.05 mg/g for a 400 mg/L initial Cr(VI) concentration. Pomegranate peel (PG) and pomegranate peel-derived biochar (PG-B) were studied by Chen et al. [108] for the removal of Cr(VI); the maximum adsorption efficiency was found to be 90.50% by PG-B in 30 min while PB demonstrated a 78.01% Cr(VI) adsorption efficiency at 60 min. These studies indicate that food waste may be used as an effective adsorbent material for the remediation of Cr(VI).

### 4.3. Industrial Waste

Industrial waste, or the leftovers from several industrial operations, is a major environmental problem that may also be a useful resource for reducing pollution. Heavy metals may be successfully extracted from wastewater using these wastes, which include fly ash, blast furnace slag, brewer’s leftover grain, and sludge [109]. More specifically, Cr(VI) may be eliminated using these industrial wastes. For example, Zafu et al. [110] used magnetically activated carbon made from brewer’s leftover grain to remove Cr(VI), reaching a maximum removal efficiency of 97.5% at the ideal pH of 2, 30 min of contact time, and 5 g/L of adsorbent. In another study, Geremias et al. [111] reported that waste grain biochar from Brewer has an adsorption capacity of 78.13 ± 0.87 mg/g for Cr(VI), after using it to adsorb Cr(VI). Brewer’s waste grain was also used by Vendruscolo et al. [112] to remove Cr(VI) by adsorption. They removed Cr(VI) from pre-treated brewery waste grain using acid, alkali, petroleum ether, and H_2_O_2_. At a pH of 2 and an adsorbent dose of 2 g/L, the percentages of Cr(VI) removed were 97%, 21%, 0.9%, and 0.5%, respectively. Zhou et al. [113] used distiller grain-prepared biochar that had been treated with phosphoric acid (H_3_PO_4_) and WPPA modification (wet process–phosphoric acid modification) to remove Cr(VI). They reported a sustained release of nutrients and a maximum remediation rate of 83.57% for chromium.

### 4.4. Forest Waste

Forestry wastes are the lignocellulosic waste that could assist in the removal of chromium and also possess numerous benefits such as, low economical value, abundant availability, high efficiency in the removal of metal ions from wastewater and the metal ions adsorbed could be converted as well as can be reused too [114,115,116,117,118]. A variety of forestry wastes, including bark and leaves, can be used to remove Cr(VI). Barbosa et al. [119] used eucalyptus residues that had been converted into cellulose macro- and nanofibers for the sorption of Cr(VI), and they reported a 54% elimination of Cr(VI). In contrast, Yang et al. [120] used pine tree (*Pinus* sp.) bark to remediate Cr(VI) and found that the bark’s maximum ability to remove Cr(VI) was 376.3 mg/g. Activated carbon prepared from *Ficus carica* leaves and modified activated carbon with ethyl diamine was utilized by Baassou et al. [121] for the adsorption of Cr(VI) and obtained maximum chromium adsorption capacities of 155.22 mg/g and 203.25 mg/g by AC-100 (activated carbon) and modified form, respectively.

## 5. Biomass-Based Adsorption Mechanisms for Cr(VI) Removal

The efficient and environmentally friendly methods of removing Cr(VI) via biomass-based adsorption are well-known, especially when they employ agricultural biowaste. Complex mechanisms including electrostatic attraction, reduction-coupled adsorption, and ion exchange are all involved in these techniques [122]. Fruit peels and other agricultural biowaste, such as crop residues, are subject to ion exchange, where Cr(VI) ions interact with functional groups such as carboxyl, hydroxyl, and amine groups on their surface. The pH of the solution has a major impact on the process of exchanging these functional groups’ cations for Cr(VI) anions [123]. The biowaste surface becomes more attractive to negatively charged Cr(VI) ions at lower pH values because of protonation, whereas deprotonation of functional groups at higher pH levels results in decreased ion exchange efficiency [124].

The efficacy of adsorption is significantly influenced by the physical characteristics of agricultural biowaste, including surface area, porosity, and particle size. Greater surface area and porosity in biowaste provide more adsorption sites; smaller particle sizes raise the surface area-to-volume ratio and enhance the biowaste’s interactions with Cr(VI) ions [125]. Furthermore, through competitive adsorption, the presence of additional ions or contaminants in the solution might influence the adsorption process and ultimately the effectiveness of Cr(VI) removal. To improve the effectiveness of agricultural biowaste-based Cr(VI) removal and make it a sustainable and ecologically friendly technique of purifying water, it is imperative to comprehend and optimize these interactions and factors [126].

## 6. Adsorption Strategies Using Waste Biomass for Cr(VI) Removal

A sustainable approach to the efficient management of generated wastes is provided by waste material biomass, such as agricultural waste and lignocellulose wastes, used as an adsorbent [127,128,129]. The removal of hexavalent chromium (Cr(VI)) from wastewater mostly depends on adsorption, which takes advantage of the ion’s surface adhesion to an adsorbent material. The raw biomass source is agricultural waste, which is selected on the basis of its desirable characteristics such as low cost, high affinity for metal, availability in large quantities, and the easy desorption of the adsorbed metal ions [130]. Several researchers reported on Cr(VI) removal through the adsorption phenomenon [130,131,132]. In one study, researchers discussed adsorbate removal efficiency or the required amounts of adsorbents during an adsorption study [133].

Different types of waste material biomass are used as adsorbents. The two main approaches that are important in the Cr(VI) remediation process are discussed below.

### 6.1. Cr(VI) Remediation Using Unmodified Biomass Adsorbents

**Figure 3:**
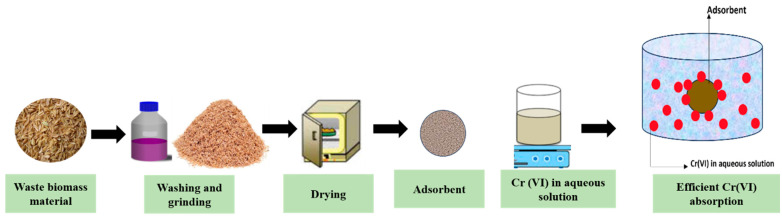
Remediation Cr(VI) using unmodified waste biomass.

A variety of biomass waste materials are used as adsorbents, after being ground and sieved, for remediating toxic Cr(VI). Several researchers have reported on the removal of Cr(VI) using waste material biomass [134,135,136,137]. This method uses unmodified waste material biomass, such as peels, leaves, etc., that have been cleaned, dried, and then ground and sieved to reduce the size of the materials, as shown in Figure 3. This provides more surface area for adsorption. For the removal of Cr(VI) with biomass waste, several studies have used this method and had successful outcomes. This is a basic technique that involves grinding, sieving, oven drying, and washing the biomass with distilled water. In one study, Verma et al. [138] studied the removal of Cr(VI) from polluted water by using coconut husks. To create a fine adsorbent, coconut husks were cut into tiny pieces, mixed, and dried in an oven for 24 h. This demonstrated the effectiveness of the technique in sequestering Cr(VI).

An orange peel, a typical fruit waste, was used as a biosorbent by Khalfaoui et al. [139] to remove Cr(VI) from water. After being cleaned of contaminants with tap water and then distilled water, the orange peels were left to dry in the sun for a few days. The dried orange peels were crushed and sieved. Orange peel powder was used as an adsorbent for each experiment. The conventional method yielded around 97.8% effective removal of Cr(VI) using orange peel as a biosorbent. To remove Cr(VI) from water containing Cr(VI) ions, Mondal et al. [140] also used a traditional method, employing mosambi peel dust (*Citrus limetta*) as an adsorbent. The removal percentage of Cr(VI) was high, indicating that the adsorbent material was successful in remediating the toxicant and demonstrated its possible application for the on-site treatment of a Cr(VI)-polluted environment.

Various researchers have reported on Cr(VI) remediation using waste biomass to remove or reduce the toxicity of Cr (Table 1).

### 6.2. Cr(VI) Remediation Using Modified Biomass Adsorbents

The use of modified biomass involves treating adsorbents differently to improve their adsorption effectiveness and effectively remediate Cr(VI). The efficient removal of pollutants from wastewater has also been accomplished with modified waste material biomass. Various chemical treatments can modify the functional groups on adsorbents and improve the active sites on their surface, increasing the adsorbents’ effectiveness [155]. Biochar is a multifaceted material with several oxygen-based functional groups on its surface that help with the adsorption and retention of pollutants such as organic materials and heavy metals. Moreover, it has a greater capacity for ion exchange, which makes it easier for it to adsorb contaminants [156]. It has been demonstrated that waste material biomass, when converted into biochar, is an effective adsorbent material for cleaning up environmental pollutants [157,158,159]. Because of its limited ability to adsorb anionic pollutants, such as phosphates and chromates [159], biochar can be modified using a variety of techniques to increase the effectiveness of adsorption [160,161]. Cr(VI) can also be remedied using activated carbon that has been activated by different activating agents [162]. Other researchers also reported the activated adsorbent materials for their applications [163,164]. These approaches may be employed in research on the remediation of Cr(VI). In one study, Chanda et al. [19] employed charcoal made from cauliflower stem waste. Using a contemporary approach, the biochar demonstrated an effective Cr(VI) adsorption capability.

**Figure 4:**
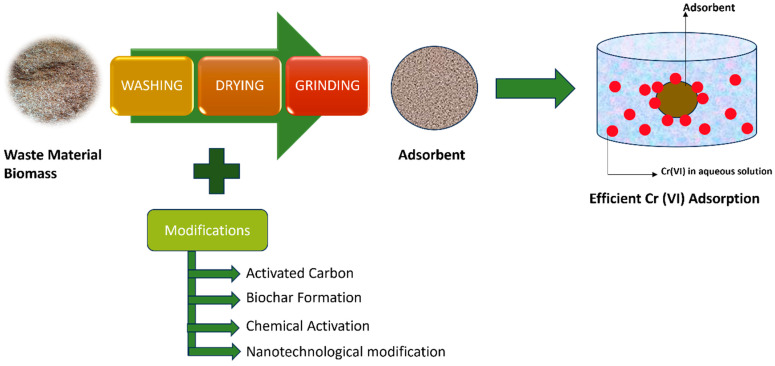
Adsorption of Cr(VI) using modified waste material biomass.

In another study, Ali et al. [2] made use of amide-modified biochar, which was produced by low-temperature pyrolysis of ground rice husks. With modified biochar, a reduction in Cr(VI) was achieved. Researchers reported on Cr(VI) removal through a biosorption process [165,166]. Other cutting-edge techniques include the extremely successful and economical technique, using carbonized rice husk modified by nano-hydroxy apatite and supported by biochar used by Zou et al. [167], in which they succeeded in removing Cr(VI) at an acidic pH. When using rice husk ash nano-silica for Cr(VI) remediation, Kandasamy et al. [168] achieved an effective removal of 88.3%. For the effective removal of Cr(VI), a variety of adsorbents with chemical modifications can also be used. After crushing banana peels, Huang et al. [169] employed chemically altered banana peel powder with 50% H_2_SO_4_ at 50 °C for 24 h. Consequently, increasing the adsorbent’s surface area led to an 80% removal rate of Cr(VI). Numerous additional investigations have also shown how well Cr(VI) is remediated with contemporary methods for altering the adsorbents made from waste material biomass (Table 2). Figure 4 showed Cr(VI) remediation process using modified waste material biomass as adsorbents.

## 7. Factors Influencing the Remediation of Cr(VI)

### 7.1. pH

Adsorption effectiveness is greatly impacted by carefully regulated operating conditions, which are necessary for optimal performance in Cr(VI) remediation utilizing waste biomass adsorbents. The selection of remediation methods, biological interactions, chemical qualities, and environmental variables all play a role in the efficacious removal of Cr(VI) from polluted settings. It is essential to comprehend these elements to create effective plans for reducing the effects of Cr(VI) pollution. For Cr(VI) to effectively adsorb on an adsorbent, the pH must be at its ideal level. A study by Khalil et al. [41] reported on the efficient removal of Cr(VI) at a pH of 5.2. Areti et al. [174] and Garg et al. [176] demonstrated that the efficient removal of Cr(VI) was obtained at a pH of 2.

### 7.2. Adsorbent Dosage

The dose of the adsorbent affects Cr(VI) ion adsorption in aquatic environments [183,184,185]. The removal of Cr(VI) is reduced at higher adsorbent doses because of the overlapping of adsorbent particles, which reduces the binding sites for Cr(VI) adsorption [41,178]. Optimization of different process parameters is required for determining the appropriate conditions and the amount of adsorbent for the effective remediation of Cr(VI) from polluted wastewater as well as for possible large-scale on-site applications.

### 7.3. Temperature

The impact of temperature on the adsorption of Cr(VI) by a mosambi peel adsorbent was investigated by Mondal et al. [140]. The researchers discovered that whereas Cr(VI) removal increased steadily from 35 to 45 °C, it declined beyond 45 °C. According to the study, the removal of Cr(VI) with a mosambi peel adsorbent is an endothermic process. The removal rate of Cr(VI) increases as a result of the higher temperature, which helps to produce more new sorption sites on the surface of adsorbents. Using powdered corn cob for the biosorption of Cr(VI) from an aqueous solution, Sallau et al. [186] similarly noted the same trend of enhanced Cr(VI) removal by raising the temperature and subsequently noted decreased removal after 80 °C. In another study, Fontecha-Cámara et al. [187] studied the endothermic nature of the adsorption process for the herbicide diuron from aqueous solutions on activated carbon fiber. They reported that the endothermic adsorptions revealed overall processes involving the combination of endothermic (probably dehydration) and exothermic steps (adsorption). In such situations, the usual van’t Hoff law cannot be applied, as the overall process involves the combination of endothermic and exothermic processes (adsorption).

### 7.4. Initial Cr(VI) Concentration and Co-Occurring Ions

A trend of increasing adsorption with a rise in the initial Cr(VI) concentration was reported by Niam et al. [188], who noted a reduction in adsorption beyond a specific point in the solution’s Cr(VI) concentration. At the lower concentration of Cr(VI), the available sites for the adsorption of Cr(VI) ions are greater; however, at a higher concentration, the adsorption sites are fewer. The presence of co-occurring ions may also affect the removal of specific metals. It was shown that the adsorption of Cr(VI) on the adsorbents might be significantly impacted by the presence of these ions [41]. According to Khalil et al. [41], sulfate ions have a greater potential to influence Cr(VI) elimination than nitrate and phosphate ions.

### 7.5. Effect of Contact Time

According to Srividya and Mohanty [189], it is essential in the chemistry of adsorption to provide the solution with enough time to reach an equilibrium condition. As the contact period rises, more vacant sites become available for adsorption, which leads to an increase in the adsorption of Cr(VI) ions on the adsorbent. At a certain moment, equilibrium is reached, indicating that all the sites are occupied at equilibrium. Nevertheless, when adsorption increases, Cr(VI) ions occupy the vacant sites [9]. Pant et al. [190] also reported a similar result, with an increment in adsorption with time and equilibrium after 180 min while using CALS for the adsorption of Cr(VI).

### 7.6. Modification Methods

The removal of Cr(VI) from aqueous solutions may be greatly improved by employing biomass waste and a variety of cutting-edge modification methods. The surface chemistry of biomass can be altered chemically by applying bases and acids, which deprotonate surface sites and introduce carboxyl and hydroxyl groups, to increase the number of functional groups available for Cr(VI) binding. Cr(VI) is converted to the less poisonous Cr(III) via metal impregnation with salts, such as iron or aluminum, improving elimination [191]. While particle size reduction improves the surface area-to-volume ratio, physical alterations, such as high-temperature heat activation, provide activated carbon or biochar with improved surface area and porosity. Adsorption is further improved by biological alterations, such as composting or microbial treatments, which provide more functional groups or enzymatic activity. Despite the possible higher costs, modified adsorbents often exhibit larger adsorption capacities, quicker kinetics, and enhanced removal efficiencies, making these procedures a sustainable and cost-effective alternative for Cr(VI) remediation [192,193].

## 8. Challenges and Future Prospects

When using biomass from agricultural waste to treat complicated industrial effluents, biosorption has proven to be an economically feasible approach for heavy metal remediation. Because of the difficulties, such as chromium’s various oxidation states, that make the management and monitoring of biosorption more difficult, biosorption is still mostly limited to laboratory settings, even though its efficacy has been well studied. The large-scale supply of biomass, its regeneration and reuse, the limited robustness of biosorbents, and the interference from co-occurring ions are practical challenges that commonly plague conventional remediation procedures, which also struggle with efficiency.

Many aspects need to be taken into account in order to overcome these obstacles and improve the practical use of biomass adsorbents for chromium (VI) remediation. Pilot-scale trials and other technical feasibility studies are necessary to move from laboratory research to real-world applications. The total efficacy of water treatment methods, such as chemical precipitation and membrane filtration, might be enhanced by being integrated with biosorption. An understanding of the environmental implications requires evaluating the sustainability of utilizing waste biomass using carbon footprint studies and life cycle assessments (LCA). Financial feasibility and ideal market sectors may be ascertained with the use of economic analyses, including cost–benefit and market potential evaluations. It is also critical to create a regulatory environment that is favorable and to guarantee worker safety while managing biomass adsorbents. Involving local communities and stakeholders may help to promote its acceptability and draw attention to social advantages such the development of jobs.

Expanding adsorption studies should be the main goal of future studies, especially when applying continuous column techniques to industrial effluents. Progress in nanomaterials, such as adding graphene or carbon nanotubes to biomass, might improve adsorption efficiency dramatically. Efficiency might be enhanced by mixing biomass with synthetic materials or modifying it with chemical agents to generate hybrid adsorbents. A deeper understanding of adsorption processes may be gained by utilizing kinetic studies and molecular dynamics simulations. Cleanup efforts might also be optimized by creating adsorbents that are self-cleaning and regenerative, and by combining adsorption with cutting-edge technologies like photocatalysis or electrochemical reduction. More efficient, sustainable, and financially feasible Cr(VI) remediation methods will be possible with the integration of green chemistry concepts and field testing. These methods will yield important information on the viability and efficacy of these strategies.

## 9. Conclusions

Waste material biomass has been shown to be quite efficient in the remediation of Cr(VI) in aquatic environments. Cr(VI) is hazardous for the overall health of our ecosystem, affecting various facets of the ecosystem. Therefore, utilizing waste biomass for the sequestration of Cr(VI) has been identified as a resilient and cost- effective technique. Adsorbents derived from waste material biomasses have been reported to be exceptionally efficient. The modification of adsorbents to enhance remediation efficiency has additionally demonstrated the potential of this technique. This approach has been identified as a potent technique in facilitating the detoxification of Cr(VI) from the environment. Therefore, the utilization of this simple, sustainable, and inexpensive strategy using waste material biomass is a promising solution for remediating a hazardous contaminant from the environment. However, further research is needed to improve the effectiveness of this environmentally friendly technology and broaden its use in the remediation of Cr(VI) from contaminated sites.

## Figures and Tables

**Table 1:** Strategy for the remediation of Cr(VI) by waste material biomass.

S. No.	Waste Material Biomass	Additional Treatment	Experimental Conditions	Adsorption Capacity (mg/g)	Removal Efficiency (%)	References
1.	Maize cob	Maize cob powdered	Batch experiment Adsorbent dose = 5.0 mg/L	-	99.1%	[141]
2.	Mango kernel	Mango kernel powdered	Temperature = 298 K Contact time = 30 min	94.8	-	[142]
3.	Raspberry canes	-	Initial concentration of Cr(VI) = 50 mg/L pH = 2	-	~95%	[143]
4.	*Sambucus nigra* leaves (Agroforestry and industrial residue)	Ground and sieved	Adsorbent dosage = 3 g/L pH = 2	-	98.2%	[144]
5.	Rice husk	Dried, crushed and sieved	pH = 5.2 Initial Cr(VI) concentration = 120 mg/L Time = 2 h	379.6	78.6%	[41]
6.	*Artocarpus heterophyllus* Lam.(Jackfruit) leaves	Dried, crushed and sieved	pH = 8, Adsorbent dose = 0.5 g/L, Time = 120 min.	0.19	95%	[145]
7.	*Moringa stenopetala* seed (MSSP) and banana peel (BPP)	Dried, ground and sieved	Contact time = 120 min Adsorbent dose = 20 g/L pH = 2 (by MSSP) pH = 4 (by BPP)	-	90.9% by MSSP, 89.6% by BPP	[146]
8.	Date palm empty fruit bunch	Dried, ground and sieved	Batch adsorption studies pH = 2 Adsorbent dose = 0.3 g Agitation speed = 100 rpm Contact time = 120 min Temperature = 30 °C	70.4	58.0%	[147]
9.	Magnolia leaf	Dried, ground, re-washed, re- dried and sieved	pH = 2 Adsorbent dose = 0.5 g Initial Cr(VI) concentration = 40 mg/L Contact time = 45 min	3.96	98.8%	[148]
10.	*Musa acuminata* Bract (MAB)	Dried, crushed and sieved	pH = 2 Adsorbent dose = 0.2 g/L	36.8	87.6%	[149]
11.	*Gliricidia sepium* leaf	Dried, powdered and sieved	pH = 2 Contact time = 120 min Biosorbent dose = 0.3 g Agitation speed = 100 rpm Initial Cr(VI) concentration = 50 mg/L	-	99.9%	[150]
12.	*Heinsia crinite* seed coat (HCSC)	Dried and powdered	pH = 2 Adsorbent dose = 0.25 g Contact time = 30 min	231.7	-	[151]
13.	Raw spent coffee waste	Washed, dried, sieved	pH = 4 Contact time = 90 min Adsorbent dose = 2.5 g/L Initial Cr(VI) concentration = 100 mg/L	42.9	-	[152]
14.	Pomegranate Peel	Powdered	pH = 1 Adsorbent dosage = 25.74 mg/g Temperature = 50 °C Initial Cr(VI) concentration = 100 mg/L	25.7	100%	[153]
15.	Banana Peel	Washed, dried, ground, sieved	_	90	~100%	[154]

**Table 2:** Effective remediation of Cr(VI) using modified waste biomass.

S. No.	Waste Material Biomass	Additional Treatment	Experimental Conditions	Adsorption Capacity (mg/g)	Removal Efficiency (%)	References
1.	Sugarcane bagasse	Schwertmannite Loaded beads of lignocellulose formed by sugarcane bagasse (~2 mm)	Batch experiment	24.6	-	[170]
2.	Garcinia kola hull particles (GK-HP)	GK-HP hydrolysed by NaOH and modified	pH = 2 Temperature = 40 °C Adsorbent dosage = 8 g/L Contact time = 60 min	-	96.2%	[42]
3.	Cauliflower stem	Phosphoric acid activated biochar (PBC-350) of cauliflower stem	Ph = 2 Shaking time = 2 h Cr(VI) concentration = 200 mg/L	64.1 by PBC-450	92% by PBC-350	[19]
4.	Wheat straw	Ball mill modified biochar of wheat straw	pH = 2 Temperature = 45 °C	52.2	100%	[171]
5.	Rice straw	Activated Carbon Formed from rice straw	pH = 3	1.48	98.9 %	[172]
6.	Banana peel	Banana peel activated (in furnace)	Adsorption time = 92 min Adsorbent dose = 1.5 g/L pH = 3 Initial Cr(VI) concentration = 38 mg/L	-	94%	[173]
7.	Banana peel + Corn cob	ZnCl_2_ impregnated biochar mixture obtained from banana peel and corn cob	pH = 2.05 Time = 34.40 min Biochar dose = 0.354 g Initial Cr(VI) concentration = 23.02 mg/L	35.8	98.9%	[174]
8.	Coconut coir	Porous biochar prepared from coconut coir	Within 24 h	40.3	~90%	[175]
9.	Walnut shells	Native Walnut shell powder (NWP), Chemically modified with alkali (AWP), Citric acid walnut shell powder (CWP) used	Batch adsorption studies pH = 2	CWP = 75.2 mg/g AWP = 69.5 mg/g NWP = 64.8 mg/g	-	[176]
10.	Tea waste	Charred tea waste (By treatment of concentrated sulphuric acid) into tea waste	pH = 2 Time = 120 min	-	81.4%	[177]
11.	Tea waste	Tea waste treated with concentrated sulphuric acid give charred tea waste then NaOH, CS_2_ gave Xanthated tea waste	Batch adsorption studies performed pH = 2 Adsorbent dose = 100 mg/L Time = 120 min	-	95.6%	[178]
12.	Tamarind fruit shells (TFS)	TFS pre-treated with phosphoric acid	Experiment in continuously mixed batch reactor (CMBR) Temperature = 25 °C Initial Cr(VI) concentration = 50 mg/L Adsorbent dosage = 12 g/L pH = 2–3	-	>95%	[179]
13.	Sugar beet bagasse (SBB)	Activated carbon formed from sugar beet bagasse	pH = 4.05 Adsorbent dosage = 1.49 g/L Initial Cr(VI) concentration = 10.13 mg/L	-	50.5%	[180]
14.	Rice husk	Parboiled Rice husk ash (RHA), Parboiled rice husk ash chemically treated (RHAH^+^)	pH = 1 Initial Cr(VI) concentration = 5 mg/L Time = 30 min	-	90.9% by RHA, 97.7% by RHAH^+^	[181]
15.	Rice husk	By low temperature pyrolysis of rice husk, Amide- modified biochar synthesized	pH = 2 Contact time = 60 min Adsorbent dosage = 2 g/L Initial Cr(VI) concentration = 100 mg/L	-	97%	[2]
16.	*Azadirachta indica* leaves	Neem leaves modified to biochar	pH = 2	58.5 mg/g	-	[182]

## Data Availability

Not applicable.
